# IRGS: an immune-related gene classifier for lung adenocarcinoma prognosis

**DOI:** 10.1186/s12967-020-02233-y

**Published:** 2020-02-04

**Authors:** Xiaoshun Shi, Ruidong Li, Xiaoying Dong, Allen Menglin Chen, Xiguang Liu, Di Lu, Siyang Feng, He Wang, Kaican Cai

**Affiliations:** 1 Department of Thoracic Surgery, Nanfang Hospital, Southern Medical University, No. 1838 of North Guangzhou Avenue, Guangzhou, 510515 People’s Republic of China; 2 Harry Perkins Institute of Medical Research, QEII Medical Centre and Centre for Medical Research, The University of Western Australia, Nedlands, WA 6009 Australia; 3 Graduate Program in Genetics, Genomics, and Bioinformatics, University of California, Riverside, CA USA; 4 Guangzhou Mendel Genomics and Medical Technology Co., Ltd., Guangzhou, 510535 China; 5 Mendel Genes Inc, Manhattan Beach, CA 90266 USA

**Keywords:** Immune gene classifier, Lung adenocarcinoma, IRGS, TCGA, Prognosis

## Abstract

**Background:**

Tumour cells interfere with normal immune functions by affecting the expression of some immune-related genes, which play roles in the prognosis of cancer patients. In recent years, immunotherapy for tumours has been widely studied, but a practical prognostic model based on immune-related genes in lung adenocarcinoma comparable to existing model has not been established and reported.

**Methods:**

We first obtained publicly accessible lung adenocarcinoma RNA expression data from The Cancer Genome Atlas (TCGA) for differential gene expression analysis and then filtered immune-related genes based on the ImmPort database. By using the lasso algorithm and multivariate Cox Proportional-Hazards (CoxPH) regression analysis, we identified candidate genes for model development and validation. The robustness of the model was further examined by comparing the model with three established gene models.

**Results:**

Gene expression data from a total of 524 lung adenocarcinoma patients from TCGA were used for model development. We identified four biomarkers (MAP3K8, CCL20, VEGFC, and ANGPTL4) that could predict overall survival in lung adenocarcinoma (HR = 1.98, 95% CI 1.48 to 2.64, P = 4.19e−06) and this model could be used as a classifier for the evaluation of low-risk and high-risk groups. This model was validated with independent microarray data and was highly comparable with previously reported gene expression signatures for lung adenocarcinoma prognosis.

**Conclusions:**

In this study, we identified a practical and robust four-gene prognostic model based on an immune gene dataset with cross-platform compatibility. This model has potential value in improving TNM staging for survival predictions in patients with lung adenocarcinoma.

**Impact:**

The study provides a method of immune relevant gene prognosis model and the identification of immune gene classifier for the prediction of lung adenocarcinoma prognosis with RNA sequencing and microarray compatibility.

## Background

The high incidence and mortality of lung cancer make this disease one of the most severe public health problems worldwide. Lung adenocarcinoma accounts for 60% of all lung cancers [1]. The TNM staging system is one of the most commonly used survival classifiers for lung cancer disease stage evaluation [2]. However, this system only considers anatomical factors and does not combine these factors with personalized genetic information or patient immune status. Therefore, individual risk stratification can only be predicted based on general populations. With the application of microarray and sequencing technology, high-throughput gene expression profiles associated with prognostic clinical data can be obtained [3], providing an opportunity for the personalized evaluation of cancer recurrence risk.

In recent years, an increasing number of studies have shown that gene expression sets are able to predict cancer patient survival, assisting decision-making regarding adjuvant chemotherapy administration. For example, chromosomal instability is a hallmark of cancer that is associated with tumour heterogeneity. Therefore, the centromere and kinetochore gene expression score (CES) signature has been used to predict the prognosis of lung cancer patients after treatment [4]. Furthermore, the discovery of genetic biomarkers has played positive roles in tailoring treatment plans and avoiding unnecessary treatments. However, current gene models, even those including up to 14 genes, have neglected clinicopathological features or are far from practical [5].

The tissue microenvironment of tumour cells plays a crucial role in the development of tumours [6]. Tumour cells can mimic the functions of immune cells to induce immunosuppression by overexpressing immune-related genes, thereby promoting the proliferation and spread of tumour cells. The acquisition of immune functions maintains tumour cells that are able to survive in the immunosuppressive microenvironment. However, the expression patterns of immune-related genes or proteins in lung adenocarcinoma and their clinical significance are still unclear. With the increased study of tumour immunotherapy, immune scoring is considered an important tumour classification method to understand immunological characteristics and predict cancer patient survival [7]. However, this scoring method only utilizes the degree of immune cell infiltration rather than whole-tumour sequencing data to reflect the overall immune function in patients.

Studying the differential expression of immune-related genes in lung cancer tissue samples is of great significance for understanding the immune microenvironment of lung cancer tissues and providing new insights to improve clinical diagnostics, prevention, patient immune status evaluation and prognosis. Based on this premise, we hypothesized that an immune-related gene expression score (IRGS) could assist the prognostic evaluation of patients with lung adenocarcinoma. Independent high-throughput data from microarray platform verified the predictive power and universality of this model. Our model can predict the prognosis of patients with lung adenocarcinoma even more precisely when combined with the traditional TNM staging system.

## Methods

### Data acquisition

In brief, data were obtained from 3 independent databases. The gene expression quantification data of RNAseq [HTSeq—Counts] for a total of 524 primary tumour and 59 normal samples from the TCGA-lung adenocarcinoma (LUAD) project were downloaded using GDCRNATools [8]. Metadata associated with the downloaded files was parsed using the tool to facilitate the integration of the count table with rows are genes and columns are samples. Trimmed mean of M values (TMM) normalization of the count data was performed using edgeR. Next, 2498 immune-associated genes were obtained from the immunology database and analysis portal (ImmPort) [https://www.immport.org/shared/genelists] on June 3, 2018 [9].

In the validation stage, we screened lung adenocarcinoma microarray data from the Gene Expression Omnibus database (GEO; http://www.ncbi.nlm.nih.gov/geo/) in June 2018 with the search term “lung adenocarcinoma”. The following exclusion criteria were applied to the microarray data: (1) containing squamous cell carcinoma, large cell lung cancer or other non-small cell lung cancer samples; (2) studies with no or insufficient clinical data; (3) only one TNM staging evaluation performed; and (4) non-primary surgical specimens. After review, GSE31210, which contained 226 lung adenocarcinoma samples and a full clinical dataset, was enrolled for validation [10].

### Development of an IRGS risk model

An immune-related prognostic model was developed by using the TCGA and ImmPort datasets. First, differentially expressed genes were identified by the limma package [11]. A false discovery rate (FDR) less than 0.01 and an absolute fold change greater than 2 were defined as the significance threshold. Thus, 3013 differentially expressed genes (1173 genes with upregulated expression and 1840 genes with downregulated expression) were filtered out. Second, 364 differentially expressed immune-related genes were filtered from 2498 genes in the ImmPort database. Last, the glmnet package [12] in R was used to implement the Lasso algorithm for gene selection. With an optimal lambda value of 0.0175 based on cross validation at which the minimal mean squared error is achieved, a total of 18 genes were further subjected to univariate and multivariate CoxPH regression analysis. Four immune-related genes (MAP3K8, CCL20, VEGFC, and ANGPTL4) were ultimately included in the risk prognosis model (risk score = − 0.434 × MAP3K8 + 0.127 × CCL20 + 0.231 × VEGFC + 0.122 × ANGPTL4). The Risk score is calculated as ∑coefficients * expression values and the median risk score is − 0.2047 for the TCGA-LUAD cohort. For Kaplan–Meier (KM) survival analysis, patients were divided into a high-risk group and a low-risk group according to the median risk score calculated by this prognostic model. Heretofore, we named this immune-related gene expression score signature the IRGS system.

### External validation of the IRGS system

Independent validation was performed by using the publicly available lung cancer dataset GSE31210 profiled by Affymetrix Human Genome U133 Plus 2.0. The GSE31210 dataset consisted of 226 lung tumour samples, of which 127 had an EGFR mutation, 20 had a KRAS mutation, 11 had an EML4-ALK fusion, and 68 were triple-negative cases. The data were normalized by the Affymetrix’s probe level normalization algorithm MAS5. We use overall survival (OS) data for survival analysis. Probes for all of the four prognostic genes in the IRGS system were able to be retrieved in the GSE31210 dataset. For genes with multiple probes, only the one with the largest variation was kept. Expression value of the selected probe for each gene was transformed to log2 scale and then the IRGS model was applied for survival prediction.

### Robustness of the IRGS system

To evaluate the performance of the IRGS signature in predicting prognosis, we compared the IRGS system with previously reported signatures including the centromere and kinetochore gene expression score (CES) (CENP-A, HJURP, MIS18B, CENP-N, CENP-L, CENP-K, ZWINT, NDC80, SPC24, SPC25, NUF2, CENP-W, CENP-U and CENP-M) [4], the three-gene prognostic classifier (STX1A, HIF1A, and CCR7) [13] and a 14-gene expression assay (BAG1, BRCA1, CDC6, CDK2AP1, ERBB3, FUT3, IL11, LCK, RND3, SH3BGR, WNT3A and the three reference genes ESD, TBP, and YAP1) [5]. Consistent with the approach used in IRGS development, Kaplan–Meier overall survival curves were plotted for patients stratified according to the median risk score calculated by these models for the TCGA dataset. Then, the specificity and sensitivity of these models were analysed to calculate the area under the ROC curve.

### In silico function analysis

To further explore the key functions of the genes in the IRGS system, we performed co-expression analysis of the four genes in the TCGA lung adenocarcinoma dataset. Protein coding genes with a Pearson correlation coefficient > 0.4 and P value < 0.01 were considered to be correlated. We sorted the LUAD RNA-seq data downloaded from the TCGA into the top and bottom quartiles of MAP3K8, CCL20, VEGFC, and ANGPTL4 expression (high and low expression, respectively) by GSEA [14] to assess the enriched pathways associated with the IRGS system.

### Statistical analysis

R (Version 3.4.3) was used for most of the bioinformatics and statistical analyses including RNAseq and microarray data normalization and transformation, differential gene expression analysis, gene coexpression analysis, CoxPH and KM survival analyses, as well as ROC analysis. The in silico pathway analysis of the IRGS system was performed using GSEA software. Two-sided P-values < 0.01 were considered statistically significant.

To rule out batch effects in TCGA data collection processing, we used the dispersion separability criterion (DSC) to measure the batch effects of TCGA-LUAD RNAseq data and didn’t observe strong batch effects (DSC < 0.5, an indicator of less serious batch effects, Additional file 1: Figure S1A). We have also compared the results of differential gene expression analyses before and after batch effect removal. A very large overlap of differential expression genes was observed (Additional file 1: Figure S1B), suggesting the batch effect small.

## Results

### A subset of immune-related genes is dysregulated in lung adenocarcinoma patients

**Fig. 1:**
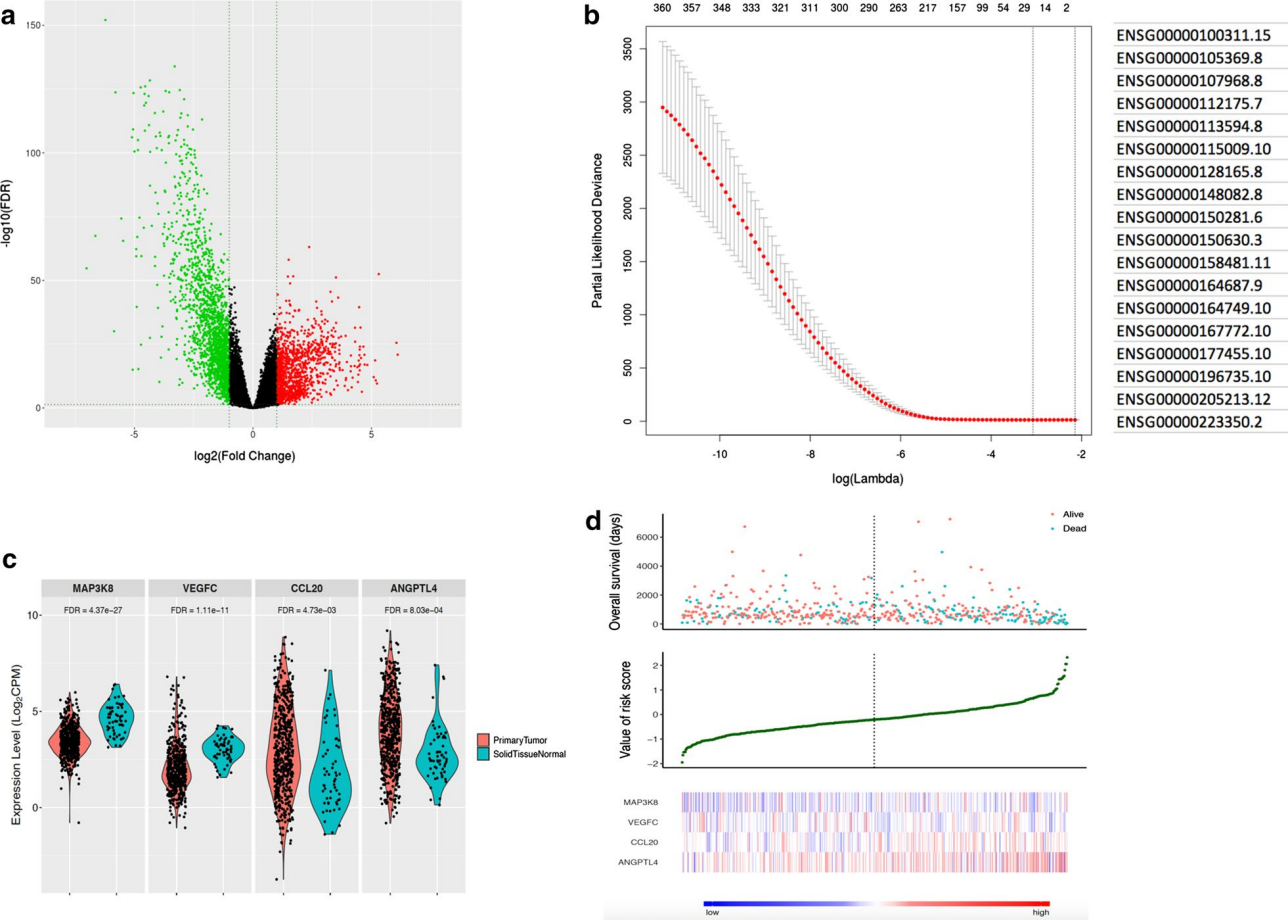
The selection of immune-related genes. **A** Differentially expressed genes in the TCGA-LUAD dataset were filtered out. **b** Immune-related genes were narrowed down by the lasso algorithm. **c** The expression of MAP3K8, CCL20, VEGFC, and ANGPTL4 in cancerous and control tissue samples is shown. **d** The distribution of the survival status, risk scores, and expression of the 4 IRGS genes in training samples are illustrated. The x axis shows the patients ranked by risk scores. High and low risk patients were separated by the dashed line

Aiming to identify immune-related genes that can classify high-risk and low-risk lung adenocarcinoma patients, we confined all the data sources in this study to RNA expression profiles of primary lung adenocarcinoma. By comparing primary tumor and solid tissue normal samples in TCGA, a total of 3013 differentially expressed genes (Additional file 2: Table S1) were identified, including 1173 up-regulated genes and 1840 down-regulated genes (Fig. 1a). The differentially expressed genes were further matched with 2498 immune-associated genes from ImmPort to generate a list of 364 candidate genes (Additional file 3: Table S2). Next, by using the lasso algorithm, the candidate genes were narrowed down to 18 with the optimal lambda value of 0.0175 (Fig. 1b). Then, univariate and multivariate Cox regression analyses were further applied. To identify key components of these candidate genes, those fit with significantly and differentially expressed between tumor and normal samples, selected as an important feature by lasso, and significant in both univariate and multivariate coxph tests were candidate genes, restricting down to 4 genes (MAP3K8, CCL20, VEGFC, and ANGPTL4) with P-values less than 0.01 as potential parameters for use in our model (Additional file 4: Table S3). Using the TCGA database, we analysed MAP3K8, CCL20, VEGFC, and ANGPTL4 gene expression levels in both normal tissue and tumour tissue samples. The four immune-associated genes displayed dysregulation in the tumour tissue samples compared with the normal tissue samples (Fig. 1c). The distribution of the survival status, risk scores, and expression of the 4 IRGS genes in training samples are illustrated in Fig. 1d. None of these genes are common driver genes in lung cancer patients. We speculate that the collaboratively abnormal expression of the immune-related genes could be regarded as an independent prognostic factor.

### Development and independent validation of IRGS

**Fig. 2:**
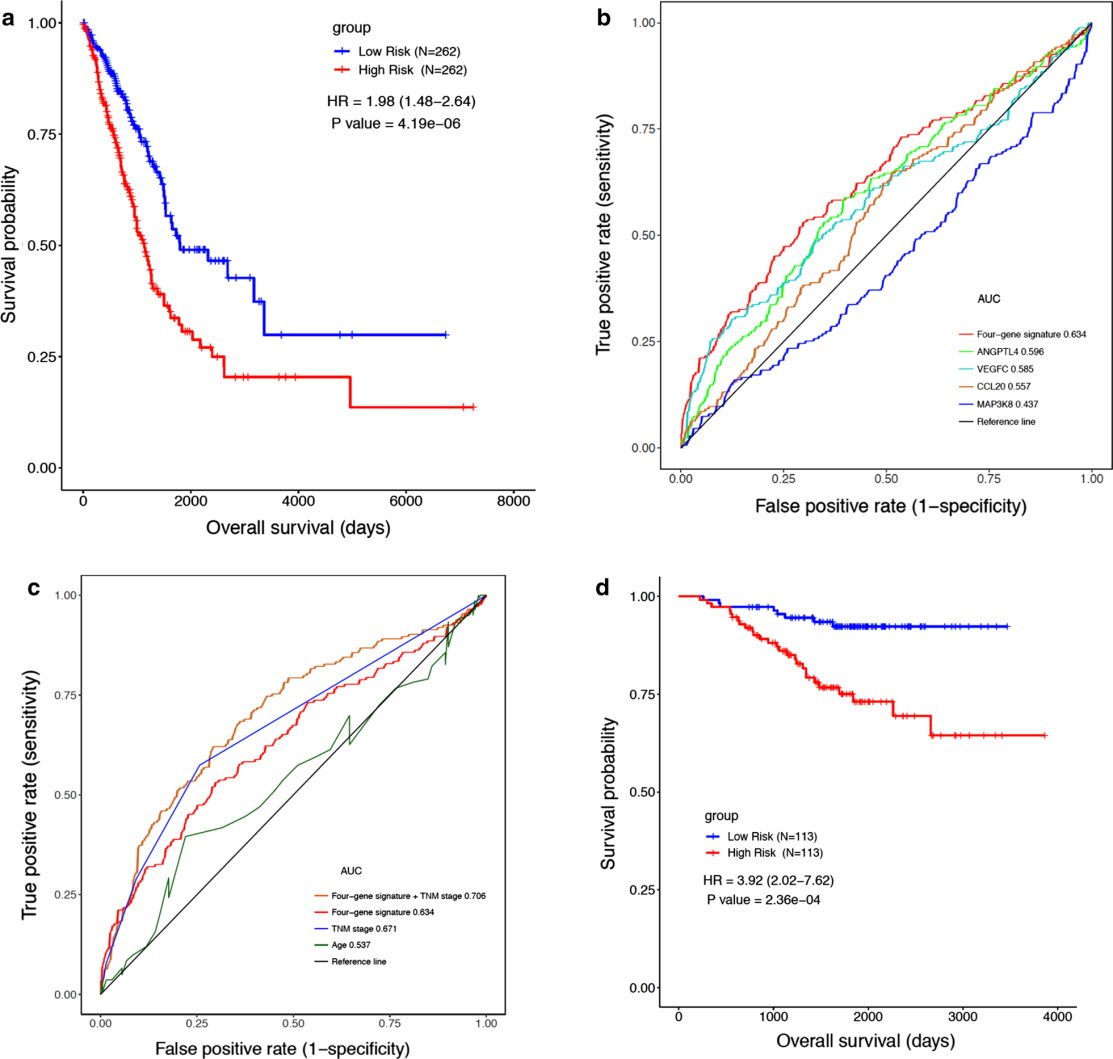
The development and validation of IRGS. **a** Kaplan–Meier analysis of overall survival in high- and low-risk groups of the lung adenocarcinoma patients in the TCGA-LUAD dataset; **b** ROC curves of the IRGS system and gene components in the system; **c** the area under the ROC curve (AUC) is given, IRGS system, TNM system, age and TNM plus the IRGS system. **d** Kaplan–Meier analysis of overall survival in high- and low-risk groups of 226 lung adenocarcinoma patients in an independent microarray dataset

Next, we enrolled the 4 immune-related genes in building a prediction model to assess the recurrence risk of lung adenocarcinoma patients. Both univariate and multivariate CoxPH survival analysis in the TCGA dataset indicated that the risk score (the minimal, maximum and median of risk score of our model in TCGA-LUAD dataset are − 1.9454, 2.3268 and − 0.2047) calculated by the IRGS model could be used as an independent prognostic factor for lung adenocarcinoma patient survival (Table 1). The median value of the risk score was then used to stratify patients; patients scoring greater than the median value were considered to have a poor prognosis, whereas patients scoring less than the median value were stratified as having a good prognosis. Kaplan–Meier survival analysis showed that the IRGS model is capable of stratifying the patients into two distinct groups with significant difference in overall survival (HR = 1.98, 95% CI 1.48 to 2.64, P = 4.19e−06); Fig. 2a). In addition to TNM staging alone in TCGA LUAD cohort (Additional file 5: Figure S2A–D), examination of our model at different stages of the lung adenocarcinoma revealed that the IRGS can separate low-risk and high-risk patients throughout TNM stages, namely stage I (HR = 2.13; CI 1.30 to 3.48; P = 2.73e−03), stage II (HR = 2.01; CI 1.22 to 3.32; P = 6.12e−03), stage III (HR = 2.62; CI 1.66 to 4.11; P = 3.32e−05), and stage IV (HR = 2.70; CI 1.02 to 7.30; P = 0.049).

**Table 1:** Univariate and multivariate Cox regression analyses of the IMAGES signature in the TCGA dataset

Variable	Univariate analysis			Multivariate analysis		
	HR	95% CI	P value	HR	95% CI	P value
Risk score	2.72	2.10–3.52	2.74e−14*	2.35	1.82–3.05	6.70e−11*
Stage	1.67	1.46–1.92	1.71e−13*	1.59	1.38–1.84	3.96e−10*
Age	1.01	0.99–1.02	0.31	–	–	–
Gender	1.07	0.80–1.43	0.63	–	–	–
Smoking status	1.03	0.89–1.18	0.71	–	–	–

To test the applicability of the IRGS system, we applied ROC analyses to the training cohort. These analyses revealed that the individual genes in the model could not function as prognostic factors; they had to be combined (Fig. 2b). Further analysis revealed that the IRGS system demonstrated a significant improvement over age or TNM staging in predicting prognosis. Moreover, the prognostic capability increased 11% when the IRGS system was combined with the traditional TNM staging system, yielding an AUC of 0.706 (Fig. 2c) and suggesting that the IRGS is a powerful tool in LUAD survival prediction.

To further verify whether the model we created can be tested by independent validation and have cross-platform universality, microarray data with clinical information were obtained from the GEO database. Following the application of the exclusion criteria, the GSE31210 microarray dataset was extracted from among 12 candidate datasets. Consistent with the training cohort results, the result of a univariate Cox regression analysis suggested that the IRGS signature was significantly associated with survival (HR = 2.11; 95% CI 1.29 to 3.46; P = 2.93e−03) in patients with lung adenocarcinoma (Additional file 6: Table S4). Kaplan–Meier analysis found that the IRGS model distinguished the high-risk group from the low-risk group, effectively differentiating the survival of the external 226 lung adenocarcinoma patients in the microarray dataset (HR = 3.92; 95% CI 2.02 to 7.62; P = 2.36e−04; Fig. 2d). Thus, we found that the IRGS signature can be used in both the TCGA RNA-sequencing data and lung microarray datasets to predict the survival of lung adenocarcinoma patients with different TNM-stage disease.

### Performance of the IRGS in predicting LUAD prognosis

**Fig. 3:**
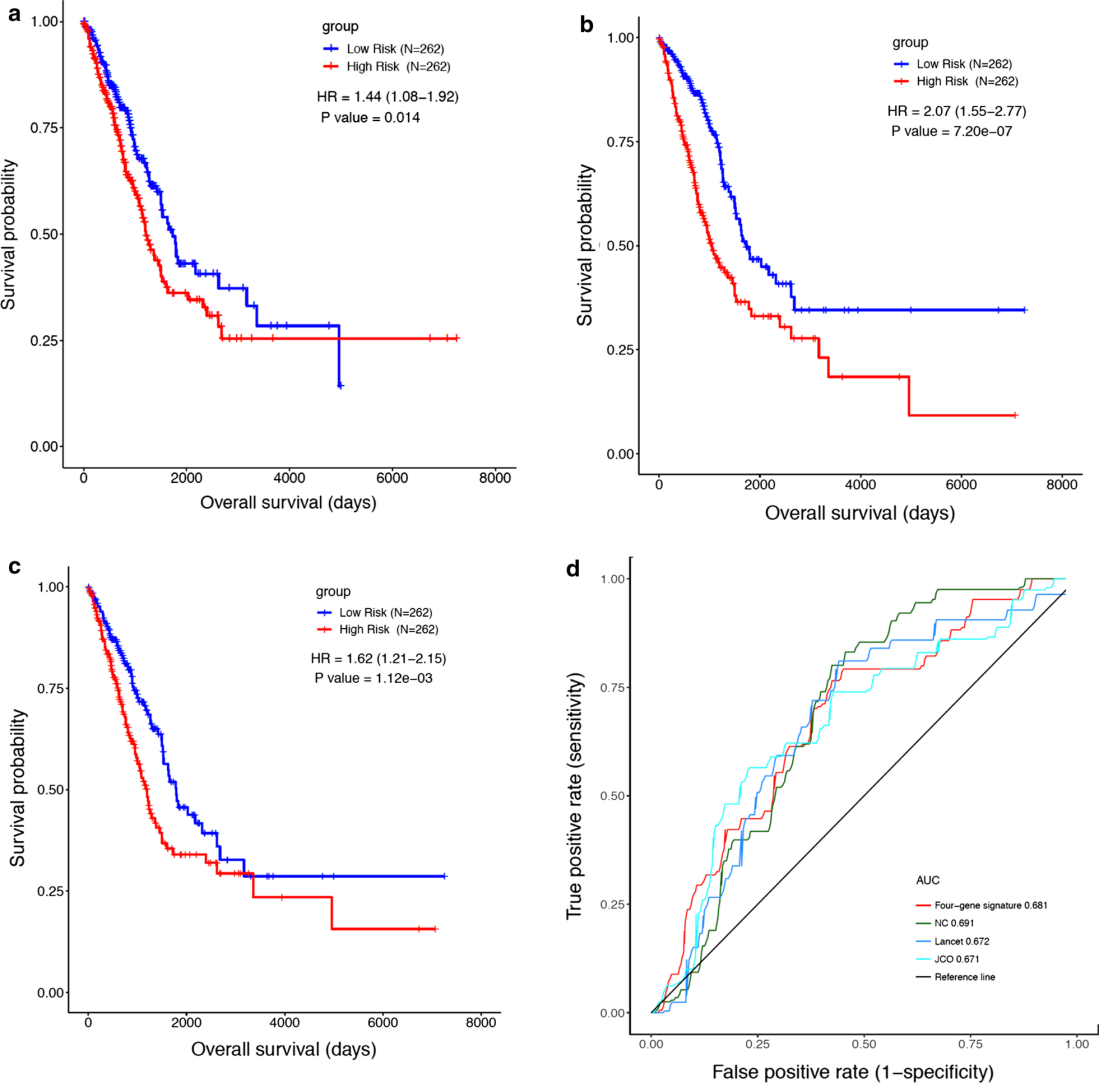
The IRGS system was compared with other gene models in the analysis of a unified RNA-sequencing dataset. **a** Kaplan–Meier curves were plotted for the TCGA LUAD dataset stratified by the three-gene classifier; **b** Kaplan–Meier curves were plotted for the TCGA LUAD dataset stratified by the CES signature; **c** Kaplan–Meier curves were plotted for the TCGA LUAD dataset stratified by the 14-gene assay; and **d** ROC curves for the IRGS signature, the three-gene classifier, the CES signature and the 14-gene practical assay were plotted

Some prognostic gene signatures for lung cancer have been reported previously, and the robustness of the IRGS was compared with the three-gene classifier, the CES signature and the 14-gene practical assay in both the TCGA RNAseq training dataset and in the independent microarray validation dataset. We first extracted a list of genes in the literature from the TCGA-LUAD dataset and submitted them to the bioinformatic pipeline used for IRGS development. Survival curves were generated (Fig. 3a–c) and showed that all three prognostic models effectively differentiated the survival of lung cancer patients (three-gene classifier: HR = 1.44, 95% CI 1.08 to 1.92, P = 0.014; the CES signature: HR = 2.07, 95% CI 1.55 to 2.77, P = 7.20e−07); and the 14-gene practical assay: HR = 1.62, 95% CI 1.21 to 2.15, P = 1.12e−03). ROC analysis was performed with the same TCGA cohort, and the AUC values were 0.602 for the three-gene classifier, 0.616 for the CES signature, and 0.598 for the 14-gene practical assay. The AUC value of the IRGS was 0.644 (Fig. 3d), leading us to conclude that the IRGS prognostic scoring system is more effective and accurate than previous gene expression signatures in predicting the survival of lung adenocarcinoma patients.

In the independent microarray validation dataset, all the three classifiers were capable of stratifying low-risk and high-risk lung adenocarcinoma patients (three-gene classifier: HR = 2.35, 95% CI 1.21 to 4.56, P = 0.015; the CES signature: HR = 4.30, 95% CI 2.22 to 8.35, P = 1.59e−04; and the 14-gene practical assay: HR = 3.80, 95% CI 1.95 to 7.37, P = 3.53e−04; Additional file 7: Figure S3A–C). The AUC values were 0.671 for the three-gene classifier, 0.691 for the CES signature, and 0.672 for the 14-gene signature, whereas the AUC value for the IRGS was 0.681 (Additional file 7: Figure S3D). Taken together, the IRGS prognostic scoring system is comparable with previous gene expression signatures in predicting the survival of lung adenocarcinoma patients using gene expression data from the microarray platform.

### Functional analysis of the IRGS signature

**Fig. 4:**
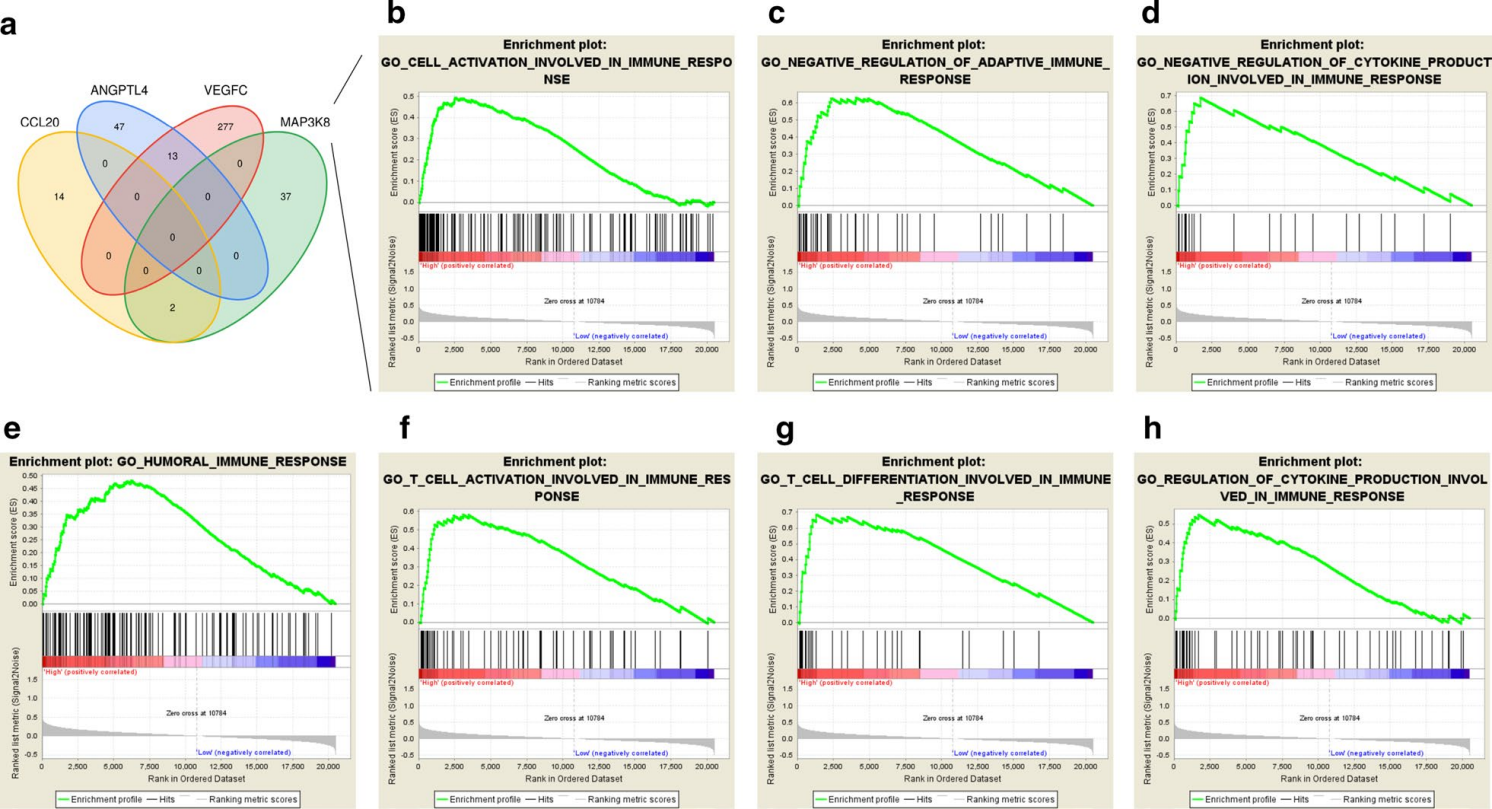
The functional analysis of the IRGS. **a** The Venn diagram indicates that each gene in the IRGS model has an individual biological function; **b**–**h** MAP3K8 and its co-expressed genes are involved in multiple immune-related responses

To further explore the core biological mechanism of our model, we performed individual co-expression analyses of the four genes with the TCGA lung adenocarcinoma RNA-sequencing dataset. Among the co-expressed genes, 39 genes were significantly associated with MAP3K8, 16 genes were significantly associated with CCL20, 290 genes were significantly associated with VEGFC, and 60 genes were significantly associated with ANGPTL4, but no common intersections were found among the co-expressed genes, suggesting that our model is the optimal gene combination (Fig. 4a). Next, we performed GSEA analysis of the 4 genes in the IRGS model (Additional file 8: Table S5). In the high CCL20 expression phenotype, regulation of immunoglobulin production, positive regulation of immunoglobulin production, positive regulation of the innate immune response, regulation of the T helper 1-type immune response, activation of the innate immune response, positive regulation of B cell-mediated immunity, and positive regulation of the immunoglobulin-mediated immune response were significantly enriched. Similarly, the pathways of T cell activation involved in the immune response, negative regulation of the adaptive immune response, negative regulation of cytokine production involved in the immune response, the humoural immune response, T cell differentiation involved in the immune response, and regulation of cytokine production involved in the immune response were found to be significantly enriched in the high MAP3K8 expression phenotype (Fig. 4b–h). Regarding the high VEGFC expression phenotype, 48 immune-associated pathways were significantly enriched (Additional file 8: Table S5), ranging from innate to adaptive immune responses. Our functional analysis indicates that genes in the IRGS system participate in a variety of immune functions in LUAD tissue.

## Discussion

Tumour staging (AJCC/UICC-TNM classification) summarizes data for the tumour burden (T), the presence of cancer cells in draining and regional lymph nodes (N), and metastatic evidence (M) to evaluate the disease stage and predict survival. However, anatomical classification provides limited information for estimating cancer patient outcomes, and the response to treatment cannot be predicted by this system. In 2005, Pages et al. showed that immune cell infiltration and the tumour microenvironment are associated with tumour prognosis [15]. Two years later, Galon et al. proposed the concept of the immunoscore [16] and described key immune components associated with cancer patient survival [17]. The immunoscore was first described as a prognostic marker for early stage (I/II) colorectal cancer patients in 2009 [18]. There have been updated results supporting the implementation of the immunoscore as a new component of a TNM immune cancer classification system [19]. These studies suggest that immunological classification may be superior to the AJCC/UICC TNM classification system alone and that introducing immune-relevant parameters as prognostic factors could be of great clinical significance. The immunoscore method is based on the evaluation of the type, density, and location of immune cells [16] and fails to comprehensively consider the balanced conditions between tumour immune functions and host immune cells, which is a balance of multiple factors such as B cell immunity, T cell immunity, immune escape, cytotoxic effect on tumor cells by the host. An average immune related gene expression from RNA sequencing in bulk could reflect the status of immune cells and tumor cells, contributing better evaluation of disease stage and prediction of survival.

Although it has been shown in different studies that a specific gene set can predict the prognosis of cancer patients [20, 21], the number of genes in a model is often beyond practical need [22] or of immune irrelevance [23], making translational feasibility low. With the development of tumour immunotherapy, the role of the immune system in the development of cancer has once again been taken into consideration. However, how immune genes in tumour cells affect cancer prognosis is still poorly understood and has not been well reported. Based on the TCGA-LUAD dataset and a meta-review of microarray data (clinical information listed in Additional file 9: Table S6 and Additional file 10: Table S7), we established and validated a prognostic model based on the expression of immune-related genes in tumour cells. Our system provides a model with high translational feasibility (current models are based on measurement of more than 10 gene expression). Secondly, the four-gene prognostic model based on an immune gene dataset is of cross-platform compatibility. A durable model between two platform with fewer genes which is comparable to the current established multiple genes model could have a better chance to translate into clinical practice, especially aiming at understanding tumor immune environment.

To increase the utility of our model, we gradually reduced the number of differentially expressed genes from 3013 to 4 (MAP3K8, CCL20, VEGFC, and ANGPTL4) in the IRGS model by using the lasso algorithm and multivariate Cox regression analysis. In the IRGS system, we postulated that high scores are associated with increased levels of immune instability and an adverse tumour microenvironment in the cancer tissue and the IRGS genes collaboratively function as an indication of a poor prognosis in lung adenocarcinoma. This system reflects that high levels of cancer cell immune instability reduce the susceptibility of cancer cells to adjuvant treatment. The IRGS system can divide lung adenocarcinoma patients into low-risk and high-risk groups, assisting clinical decision-making by increasing the precision of tumour staging and risk stratification. Although current models are able to distinguish high-risk and low-risk patient groups [4, 5, 13], the IRGS model has a better discriminating power in RNA sequencing dataset, and the IRGS combined with TNM staging can increase the predictive capacity.

Our functional analysis suggests that the genes in the IRGS model are widely involved in the immunological process. The inflammatory process is essential in the formation and development of cancer and may be involved in tumour growth and metastasis [24]. MAP3K8 (Serine/threonine protein kinase tumour progression locus 2) has been widely reported to be an important signal for inflammatory mediators, and mutational activation of MAP3K8 may be involved in the formation of lung cancer [25]. Su et al. showed that miRNAs might participate in lung cancer progression by regulating MAP3K8 [26]. The chemokine CCL20 is abnormally expressed in non-small cell lung cancer and plays important roles in tumour cell growth, invasion and metastasis [27]. For example, the expression of CCL20 in lung cancer cells is higher in patients with advanced lung cancer than in patients with early-stage lung cancer, and this high expression is associated with a poor prognosis. Bao et al. reported that the CCL20 gene may be a prognostic risk factor or protective factor for lung adenocarcinoma [28]. These observations mean that the unstable expression of these genes can cause different oncological outcomes. VEGF-C has been widely reported in lung cancer and is involved in lung cancer tumourigenesis and associated with lymphatic metastasis; it can be used as a biomarker for evaluating recurrence and prognosis in non-small cell lung cancer [29]. ANGPTL4 (Tumour cell-derived human angiopoietin-like protein 4) can damage vascular endothelial cell junctions, increase pulmonary capillary permeability, and promote the process of tumour cells protruding through the vascular endothelium. It has also been found to be involved in the growth and metastasis of lung cancer [30].

We present an IRGS prognostic model based on a second-generation sequencing technique on tumor samples, providing an efficient assessment of the overall tumour immune status. We noticed that there are some limitations in the current TCGA based LUAD survival model. We understand that there is a need to investigate the lung adenocarcinoma prognostic model based on its intrinsic heterogeneity. However, the current RNA sequencing databases underreported pathological details. Another scenario is that comorbidities have a major impact on the treatment of lung cancer and have proven to be associated with lung cancer survival [31]. Based on current databases, the data were obtained from operable samples (mainly stage I–III), making survival model as a potential tool for early intervention after surgery. Therefore, the use of these models on advance lung adenocarcinoma is limited. To solve the problems as mentioned above, separate databases built by corresponding samples are needed in further studies. We will include these factors in future model development if detailed information were provided from the published database and will focus on these issues at the time of building our inhouse database.

## Conclusion

In summary, we identified and validated an immune-related gene expression score, which can be used as an independent prognostic signature in evaluating the survival of patients with lung adenocarcinoma. The IRGS signature was successfully validated with independent microarray data and indicated to be more efficient than existing lung cancer mRNA signatures. Further multicentre prospective validation in clinical trials is needed.

## Supplementary information

**Additional file 1: Figure S1.** The evaluation of batch effects in TCGA data processing. (A) a dispersion separability criterion did not reveal a strong batch effects; (B) A venm diagram showed that a large overlap of differential expression genes exits.

**Additional file 2: Table S1.** Differentially expressed genes in the TCGA-LUAD dataset.

**Additional file 3: Table S2.** Candidate immune-related genes matched with differentially expressed genes in the TCGA dataset.

**Additional file 4: Table S3.** Univariate and multivariate analyses of candidate genes for IRGS model development.

**Additional file 5: Figure S2.** Survival analysis of TCGA LUAD data by TNM staging. (A) Kaplan–Meier curves were plotted for the TCGA LUAD dataset stratified by T stage; (B) Kaplan–Meier curves were plotted for the TCGA LUAD dataset stratified by N stage; (C) Kaplan–Meier curves were plotted for the TCGA LUAD dataset stratified by M stage; and (D) Kaplan–Meier curves were plotted for the TCGA LUAD dataset stratified by TNM stage.

**Additional file 6: Table S4.** Univariate and multivariate Cox regression analyses of the IRGS signature in the independent microarray dataset.

**Additional file 7: Figure S3.** The IRGS system was compared with other gene models in GSE31210 dataset. (A) Kaplan–Meier curves were plotted for the GSE31210 dataset stratified by the three-gene classifier; (B) Kaplan–Meier curves were plotted for the GSE31210 dataset stratified by the CES signature; (C) Kaplan–Meier curves were plotted for the GSE31210 dataset stratified by the 14-gene assay; and (D) ROC curves for the IRGS signature, the three-gene classifier, the CES signature and the 14-gene practical assay in GSE31210 dataset were plotted.

**Additional file 8: Table S5.** Significantly enriched immune pathways of gene in the IRGS model.

**Additional file 9: Table S6.** Clinical information of TCGA LUAD data. Retrieved from the GDC website (TCGA-LUAD: https://portal.gdc.cancer.gov/projects/TCGA-LUAD).

**Additional file 10: Table S7.** Clinical information of GSE31210 data. Retrieved from GEO website (https://www.ncbi.nlm.nih.gov/geo/query/acc.cgi?acc=GSE31210).

## Data Availability

All analysed data are accessible online, and the results of this article are included within the article as well as in additional files. R code is available upon request.

## Abbreviations

AUC: Area under the curve
CES: The centromere and kinetochore gene expression score
CI: Confidence interval
DSC: Dispersion separability criterion
GEO: Gene Expression Omnibus
GO: Gene ontology
HR: Hazard ratio
IRGS: Immune-related gene expression score
KEGG: Kyoto Encyclopedia of Genes and Genomes
LUAD: Lung adenocarcinoma
ROC: Receiver operating characteristic
OS: Overall survival
TCGA: The Cancer Genome Atlas
TNM: Tumour, node, metastasis
qRT-PCR: Quantitative real-time polymerase chain reaction

## References

[1] Behera M, Owonikoko TK, Gal AA, Steuer CE, Kim S, Pillai RN, Khuri FR, Ramalingam SS, Sica GL (2016). Lung adenocarcinoma staging using the 2011 IASLC/ATS/ERS classification: a pooled analysis of adenocarcinoma in situ and minimally invasive adenocarcinoma. Clin Lung Cancer.

[2] Chansky K, Detterbeck FC, Nicholson AG, Rusch VW, Vallieres E, Groome P, Kennedy C, Krasnik M, Peake M, Shemanski L (2017). The IASLC lung cancer staging project: external validation of the revision of the TNM stage groupings in the eighth edition of the TNM classification of lung cancer. J Thorac Oncol..

[3] Shi X, Tan H, Le X, Xian H, Li X, Huang K, Luo VY, Liu Y, Wu Z, Mo H (2018). An expression signature model to predict lung adenocarcinoma-specific survival. Cancer Manag Res.

[4] Zhang W, Mao JH, Zhu W, Jain AK, Liu K, Brown JB, Karpen GH (2016). Centromere and kinetochore gene misexpression predicts cancer patient survival and response to radiotherapy and chemotherapy. Nat Commun.

[5] Kratz JR, He J, Van Den Eeden SK, Zhu ZH, Gao W, Pham PT, Mulvihill MS, Ziaei F, Zhang H, Su B (2012). A practical molecular assay to predict survival in resected non-squamous, non-small-cell lung cancer: development and international validation studies. Lancet.

[6] Quail DF, Joyce JA (2013). Microenvironmental regulation of tumor progression and metastasis. Nat Med.

[7] Fridman WH, Pages F, Sautes-Fridman C, Galon J (2012). The immune contexture in human tumours: impact on clinical outcome. Nat Rev Cancer.

[8] Li R, Qu H, Wang S, Wei J, Zhang L, Ma R, Lu J, Zhu J, Zhong WD, Jia Z (2018). GDCRNATOOLS: an R/Bioconductor package for integrative analysis of lncRNA, miRNA and mRNA data in GDC. Bioinformatics.

[9] Bhattacharya S, Andorf S, Gomes L, Dunn P, Schaefer H, Pontius J, Berger P, Desborough V, Smith T, Campbell J (2014). ImmPort: disseminating data to the public for the future of immunology. Immunol Res.

[10] Okayama H, Kohno T, Ishii Y, Shimada Y, Shiraishi K, Iwakawa R, Furuta K, Tsuta K, Shibata T, Yamamoto S (2012). Identification of genes upregulated in ALK-positive and EGFR/KRAS/ALK-negative lung adenocarcinomas. Cancer Res.

[11] Ritchie ME, Phipson B, Wu D, Hu Y, Law CW, Shi W, Smyth GK (2015). Limma powers differential expression analyses for RNA-sequencing and microarray studies. Nucleic Acids Res.

[12] Friedman J, Hastie T, Tibshirani R (2010). Regularization paths for generalized linear models via coordinate descent. J Stat Softw.

[13] Lau SK, Boutros PC, Pintilie M, Blackhall FH, Zhu CQ, Strumpf D, Johnston MR, Darling G, Keshavjee S, Waddell TK (2007). Three-gene prognostic classifier for early-stage non small-cell lung cancer. J Clin Oncol.

[14] Subramanian A, Tamayo P, Mootha VK, Mukherjee S, Ebert BL, Gillette MA, Paulovich A, Pomeroy SL, Golub TR, Lander ES (2005). Gene set enrichment analysis: a knowledge-based approach for interpreting genome-wide expression profiles. Proc Natl Acad Sci U S A.

[15] Pages F, Berger A, Camus M, Sanchez-Cabo F, Costes A, Molidor R, Mlecnik B, Kirilovsky A, Nilsson M, Damotte D (2005). Effector memory T cells, early metastasis, and survival in colorectal cancer. N Engl J Med.

[16] Galon J, Costes A, Sanchez-Cabo F, Kirilovsky A, Mlecnik B, Lagorce-Pages C, Tosolini M, Camus M, Berger A, Wind P (2006). Type, density, and location of immune cells within human colorectal tumors predict clinical outcome. Science.

[17] Galon J, Fridman WH, Pages F (2007). The adaptive immunologic microenvironment in colorectal cancer: a novel perspective. Cancer Res.

[18] Pages F, Kirilovsky A, Mlecnik B, Asslaber M, Tosolini M, Bindea G, Lagorce C, Wind P, Marliot F, Bruneval P (2009). In situ cytotoxic and memory T cells predict outcome in patients with early-stage colorectal cancer. J Clin Oncol.

[19] Pages F, Mlecnik B, Marliot F, Bindea G, Ou FS, Bifulco C, Lugli A, Zlobec I, Rau TT, Berger MD (2018). International validation of the consensus immunoscore for the classification of colon cancer: a prognostic and accuracy study. Lancet.

[20] Fatai AA, Gamieldien J (2018). A 35-gene signature discriminates between rapidly- and slowly-progressing glioblastoma multiforme and predicts survival in known subtypes of the cancer. BMC Cancer.

[21] Xu W, Jia G, Davie JR, Murphy L, Kratzke R, Banerji S (2016). A 10-gene yin yang expression ratio signature for stage IA and IB non-small cell lung cancer. J Thorac Oncol.

[22] Song Q, Shang J, Yang Z, Zhang L, Zhang C, Chen J, Wu X (2019). Identification of an immune signature predicting prognosis risk of patients in lung adenocarcinoma. J Transl Med.

[23] Zhao K, Li Z, Tian H (2018). Twenty-gene-based prognostic model predicts lung adenocarcinoma survival. OncoTargets Ther.

[24] Coussens LM, Werb Z (2002). Inflammation and cancer. Nature.

[25] Clark AM, Reynolds SH, Anderson M, Wiest JS (2004). Mutational activation of the MAP3K8 protooncogene in lung cancer. Genes Chromosomes Cancer.

[26] Su L, Li N, Huo X (2015). Mining featured micro ribonucleic acids associated with lung cancer based on bioinformatics. Thorac Cancer.

[27] Wang B, Shi L, Sun X, Wang L, Wang X, Chen C (2016). Production of CCL20 from lung cancer cells induces the cell migration and proliferation through PI3K pathway. J Cell Mol Med.

[28] Bao L, Zhang Y, Wang J, Wang H, Dong N, Su X, Xu M, Wang X (2016). Variations of chromosome 2 gene expressions among patients with lung cancer or non-cancer. Cell Biol Toxicol.

[29] Yang Y, Maimaitiyiming X, Jin C, Ahan N, Guo R, Peng C (2015). Influence of heparanase and VEGF-C mRNA expressions in lung cancer. Indian J Surg.

[30] Zhu X, Guo X, Wu S, Wei L (2016). ANGPTL4 Correlates with NSCLC progression and regulates epithelial–mesenchymal transition via ERK pathway. Lung.

[31] Gould MK, Munoz-Plaza CE, Hahn EE, Lee JS, Parry C, Shen E (2017). Comorbidity profiles and their effect on treatment selection and survival among patients with lung cancer. Ann Am Thorac Soc.

