# Supplementary figures and images for: IRGS: an immune-related gene classifier for lung adenocarcinoma prognosis
Source: J Transl Med. 2020 Feb 4;18:55. doi: 10.1186/s12967-020-02233-y (PMC7001261; doi:10.1186/s12967-020-02233-y)

Additional file 1: Figure S1.

**A**

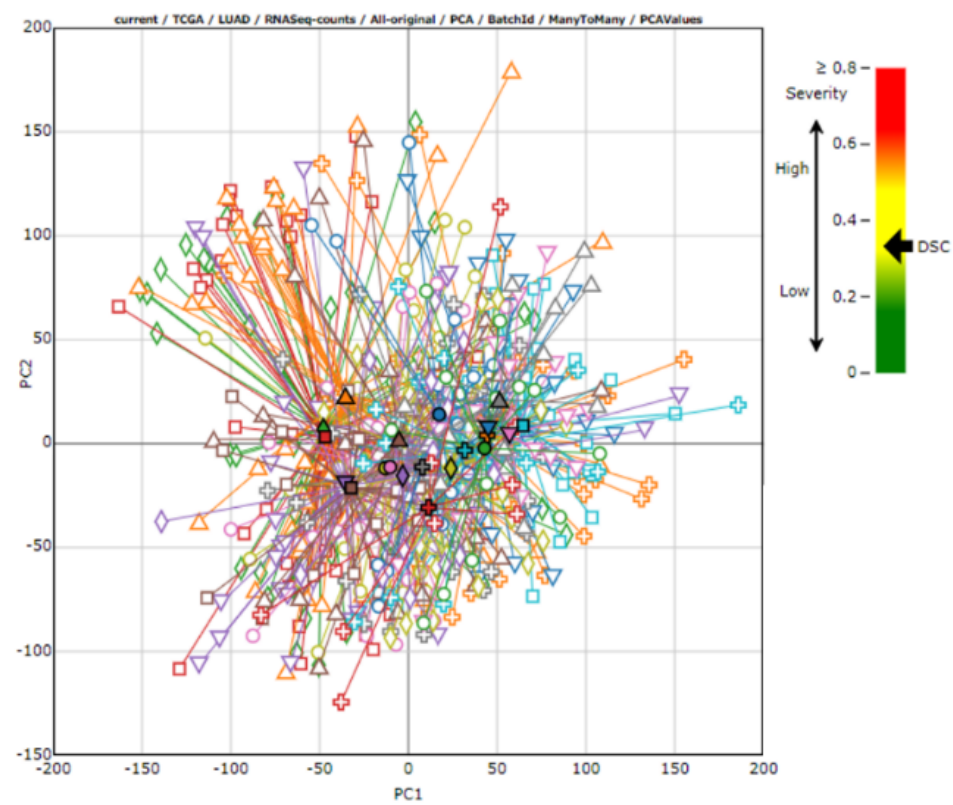

**B**

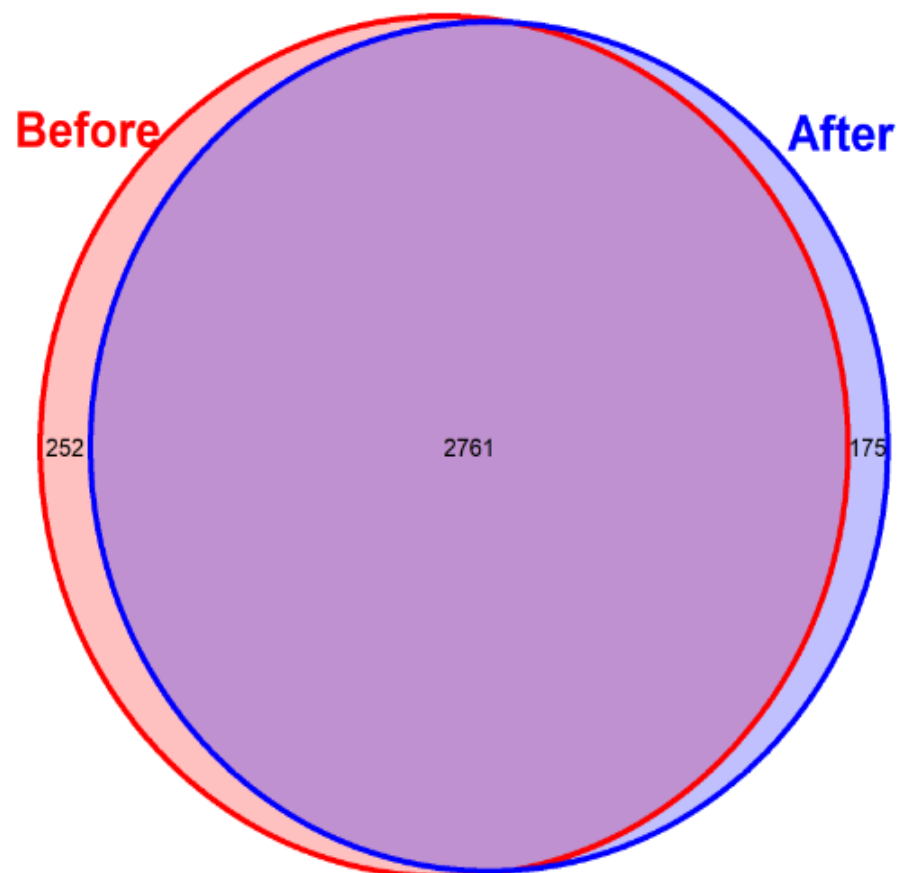

Supplement: Supplementary file 1 — Additional file 1: Figure S1. The evaluation of batch effects in TCGA data processing. (A) a dispersion separability criterion did not reveal a strong batch effects; (B) A venm diagram showed that a large overlap of differential expression genes exits. [file 12967_2020_2233_MOESM1_ESM.pdf]

Additional file 5: Figure S2.

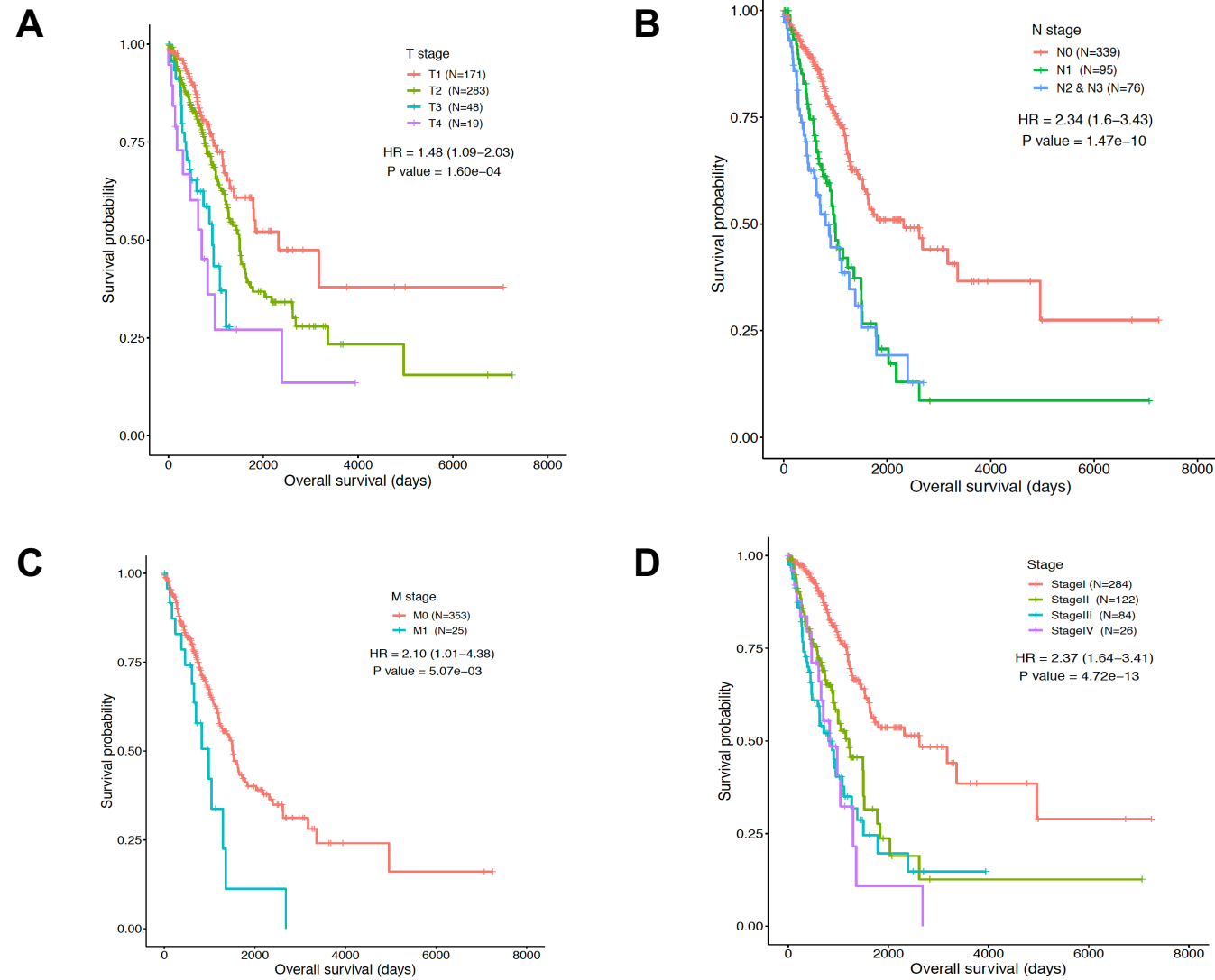

Supplement: Supplementary file 5 — Additional file 5: Figure S2. Survival analysis of TCGA LUAD data by TNM staging. (A) Kaplan–Meier curves were plotted for the TCGA LUAD dataset stratified by T stage; (B) Kaplan–Meier curves were plotted for the TCGA LUAD dataset stratified by N stage; (C) Kaplan–Meier curves were plotted for the TCGA LUAD dataset stratified by M stage; and (D) Kaplan–Meier curves were plotted for the TCGA LUAD dataset stratified by TNM stage. [file 12967_2020_2233_MOESM5_ESM.pdf]

Additional file 7: Figure S3.

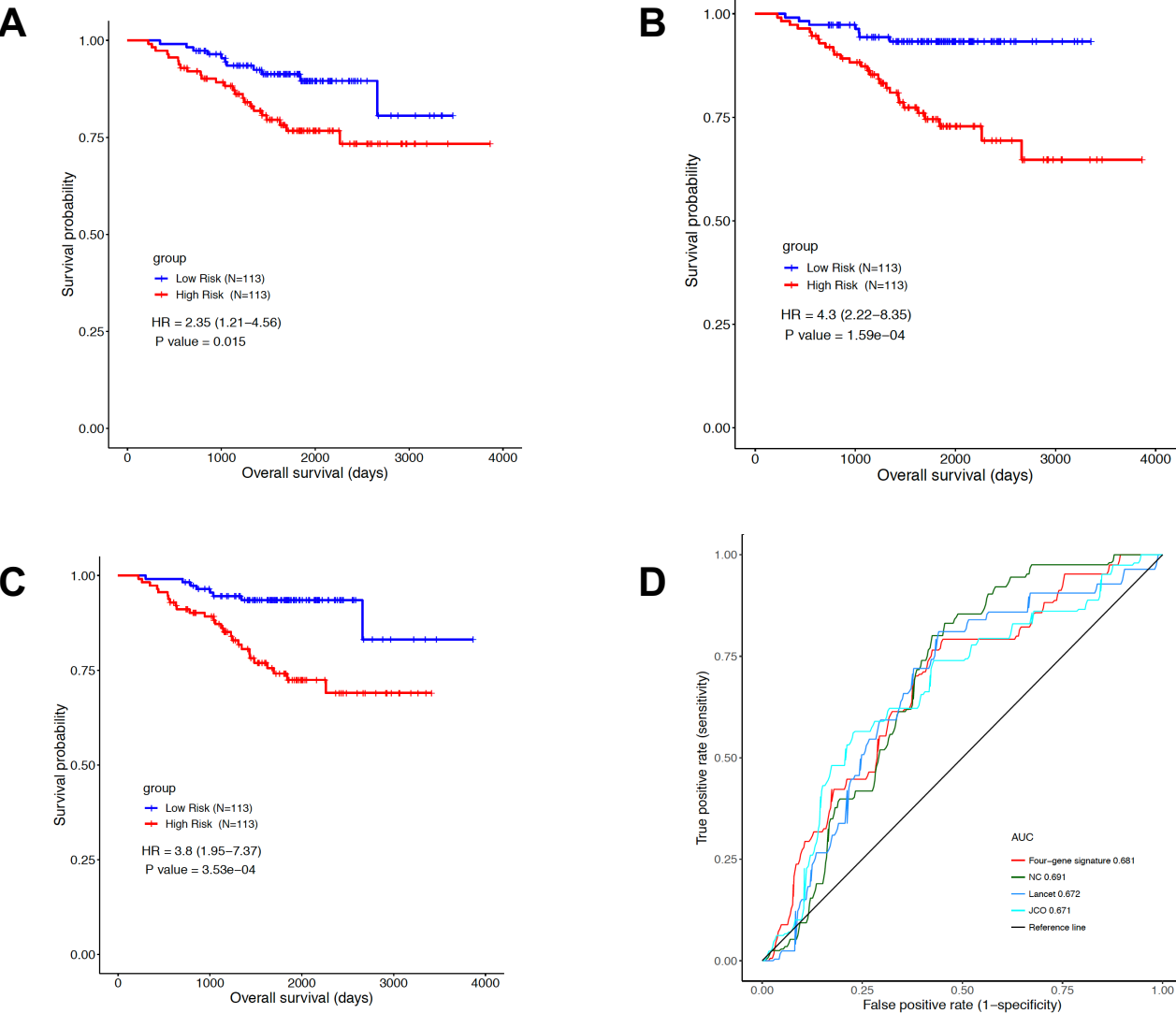

Supplement: Supplementary file 7 — Additional file 7: Figure S3. The IRGS system was compared with other gene models in GSE31210 dataset. (A) Kaplan–Meier curves were plotted for the GSE31210 dataset stratified by the three-gene classifier; (B) Kaplan–Meier curves were plotted for the GSE31210 dataset stratified by the CES signature; (C) Kaplan–Meier curves were plotted for the GSE31210 dataset stratified by the 14-gene assay; and (D) ROC curves for the IRGS signature, the three-gene classifier, the CES signature and the 14-gene practical assay in GSE31210 dataset were plotted. [file 12967_2020_2233_MOESM7_ESM.pdf]
