# Supplementary material for: IRGS: an immune-related gene classifier for lung adenocarcinoma prognosis
Source: J Transl Med. 2020 Feb 4;18:55. doi: 10.1186/s12967-020-02233-y (PMC7001261; doi:10.1186/s12967-020-02233-y)
Supplement: Supplementary file 6 — Additional file 6: Table S4. Univariate and multivariate Cox regression analyses of the IRGS signature in the independent microarray dataset. [file 12967_2020_2233_MOESM6_ESM.doc]

**Additional file: Table S4  Univariate and multivariate Cox regression analyses of the IMAGES signature in the independent microarray dataset**

| Variable | Univariate analysis | | | Multivariate analysis | | |
| --- | --- | --- | --- | --- | --- | --- |
|  | HR | 95% CI | p value | HR | 95% CI | p value |
| Stage | 4.23 | 2.17-8.24 | 2.17e-05* | 3.42 | 1.67-7.04 | 8.19e-04* |
| Risk score | 2.11 | 1.29-3.46 | 2.93e-03* | 1.52 | 0.89-2.59 | 0.12 |
| Smoking status | 1.64 | 0.84-3.20 | 0.149 | - | - | - |
| Sex | 0.66 | 0.34-1.28 | 0.218 | - | - | - |
| Age | 1.03 | 0.98-1.08 | 0.306 | - | - | - |
